# Marked Elevation of Carcinoembryonic Antigen Without an Identified Primary Gastrointestinal Tumor

**DOI:** 10.7759/cureus.20621

**Published:** 2021-12-22

**Authors:** Joshua K Salabei, Dhaval Upadhyay, Asad Haider, Anthony Nanajian, Leora Frimer, Kipson Charles, Zeeshan H Ismail, Selina Imboywa, Awaad Khan, Nundia Louis, Uma G Iyer

**Affiliations:** 1 Internal Medicine, University of Central Florida College of Medicine, Graduate Medical Education/North Florida Regional Medical Center, Gainesville, USA; 2 Internal Medicine, University of Central Florida College of Medicine, Gainesville, USA

**Keywords:** immunohistochemistry staining, metastatic adenocarcinoma of unknown primary, colorectal, cea, carcinoembryonic antigen

## Abstract

Whether profound carcinoembryonic antigen (CEA) elevations, such as > 20 times the upper limit of normal, are of diagnostic use remain unknown. Herein, we present a case of a 55-year-old female with profound serum CEA elevation and multiple pelvic masses but with no evidence of a primary gastrointestinal tumor following upper endoscopy and colonoscopy. Subsequent immunostaining of resected pelvic masses confirmed adenocarcinoma of colorectal origin. This case report highlights the possible diagnostic role of profound CEA elevation, particularly in cases of unknown primary tumors.

## Introduction

Since serum carcinoembryonic antigen (CEA) can be falsely elevated in non-malignant conditions such as chronic obstructive pulmonary disease, peptic ulcer disease, gastritis, and diabetes, it is generally considered nondiagnostic [1,2]. However, CEA levels can be used to monitor disease response to therapy if elevated pre-treatment. Whether CEA has additional utility in cases where profound elevations are observed, such as > 20 times the upper limit of normal, remains unclear. Herein, we present a case of a 55-year-old female who presented with abdominal pain and was noted to have profound serum CEA elevation on routine laboratory testing. An abdominal computed tomography (CT) showed multiple pelvic masses. Upper endoscopy and colonoscopy did not show a primary gastrointestinal (GI) tumor, although subsequent immunohistological analysis of resected pelvic masses was consistent with a primary tumor of colorectal origin. Her case highlights a possible role of profound CEA elevation in patients with metastasis in the absence of a primary GI tumor.

## Case presentation

A 55-year-old female with multiple comorbidities including hypothyroidism, diabetes type II, and morbid obesity presented to our facility complaining of upper and lower back/pelvic pain for one week. On presentation, her pain was 10/10 in severity without any associated symptoms of lumbar radiculopathy, urinary or fecal incontinence, or saddle paresthesia. She had not experienced any weight loss, night sweats, or fever. Physical exam was significant for tenderness to palpation on multiple locations on the thoracic and lumbar areas, and she was also noted to have a large palpable pelvic mass on abdominal exam. Initial studies included an unremarkable complete blood count, electrolytes, and thyroid-stimulating hormone (TSH). Tumor markers were significant for normal CA 125 and 19-9 but markedly elevated CEA (Table 1).

**Table 1:** Pertinent laboratory data at the time of presentation TSH; thyroid-stimulating hormone, CEA; carcinoembryonic antigen, CA 19-9; cancer antigen 19-9, CA 125; cancer antigen 125

Labs	Levels on admission	Normal range
White blood cells	10.8	(4.5-11.0 thousand/mm^3^)
Hemoglobin	12.1	(12.0-15.0 g/dL)
Hematocrit	38.1	(35.0-49.0%)
Platelet count	370	(150-450 thousand/mm^3^)
Creatinine	1.22	(0.60-1.30 mg/dL)
Glucose	168	(74-106 mg/dL)
Calcium	9.0	(8.5-10.1 mg/dL)
Aspartate aminotransferase	40	(15-37 U/L)
Alanine aminotransferase	16	(12-78 U/L)
Alkaline phosphatase	277	(46-116 U/L)
Total bilirubin	0.6	(0.2-1.0 mg/dL)
Creatinine kinase	227	(26-192 U/L)
TSH	3.21	(0.358-3.740 µIU/mL)
CEA	340.8	(0.0-3.0 ng/mL)
CA 19-9	24.1	(0.0-30.9 U/mL)
CA 125	5.9	(1.5-35 U/mL)

**Figure 1:**
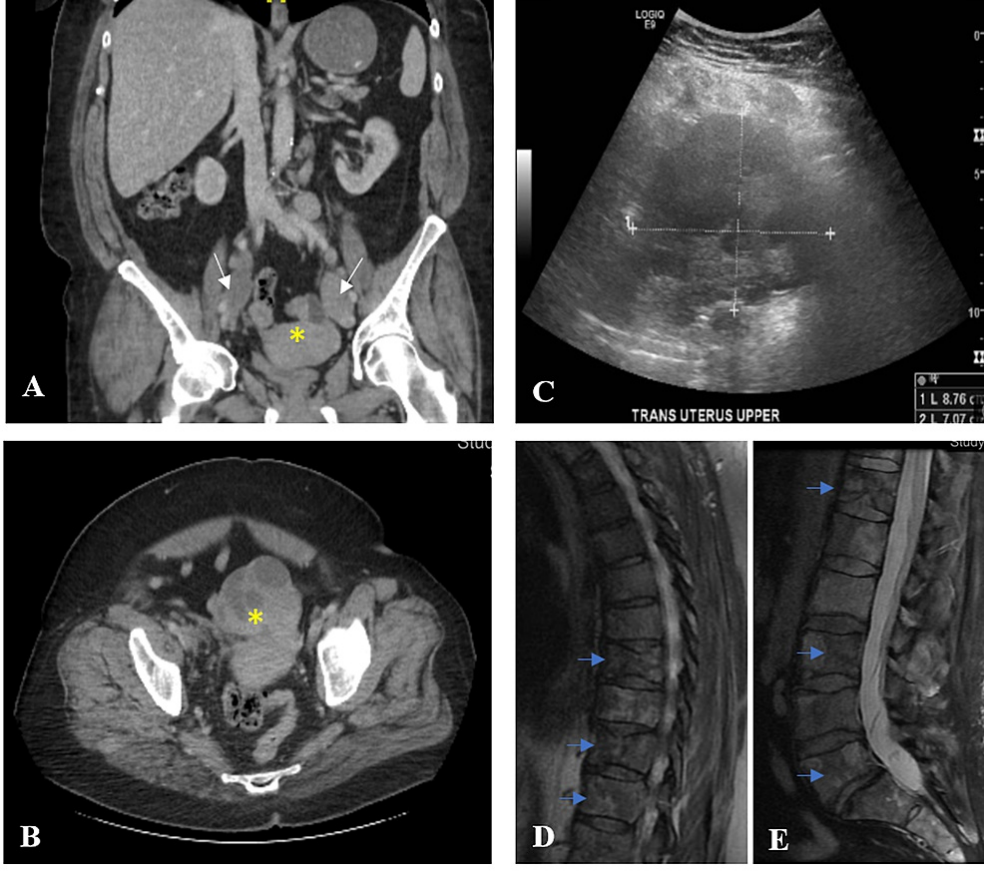
Representative CT images of pelvic mass and iliac adenopathy, and MRI showing metastasis to the spine Coronal (A) and transverse (B) views of abdomen/pelvic CT. White arrows indicate bilateral adenopathy; yellow asterisks indicate a large pelvic mass measuring 10 × 6 × 8 cm. (C) Pelvic mass, superior to the uterus, re-demonstrated on transvaginal pelvic ultrasound. MRI of the spine shows pathologic compression fractures with extensive and diffuse osseous metastatic disease (blue arrows) involving the thoracic (D) and lumbar (E) vertebrae. CT, computed tomography; MRI, magnetic resonance imaging.

A CT scan of her abdomen/pelvis showed a nodular soft tissue density along the right iliac chain measuring 8 × 3 cm, large pelvic mass measuring 10 × 6 × 8 cm, and left iliac chain adenopathy measuring 6 × 3 × 3 cm. On transvaginal pelvic ultrasound, a mass in the superior part of the uterus measuring 6.2 × 7.3 × 8.8 cm was noted (Figures 1A-1C).

**Figure 2:**
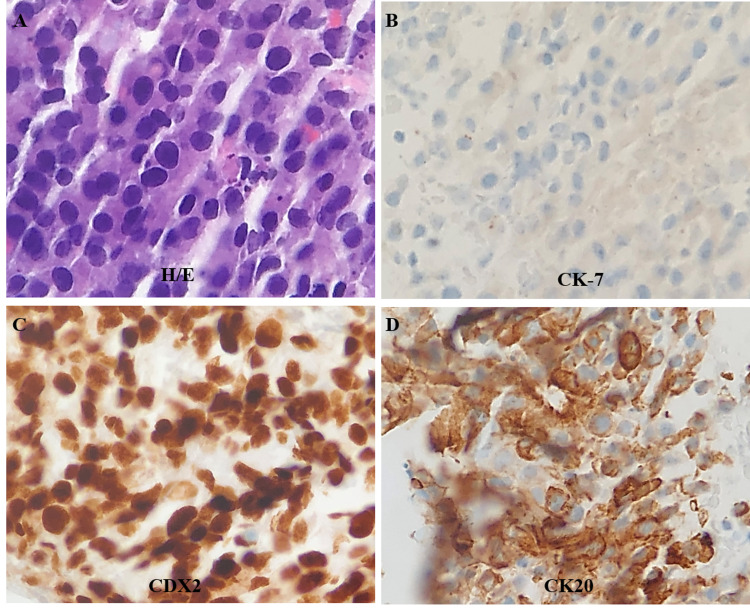
Representative images of immunostained specimen biopsied from retroperitoneal lymph nodes Immunohistochemistry. Hematoxylin and eosin (A), CK-7 negative (B), diffuse and strong immunoreactivity in the cytoplasm of cancer cells for CDX-2 (C) and CK-20 (D). Magnification x40. CK-7, cytokeratin 7; CK-20, cytokeratin 20; CDX2, caudal type homeobox 2

No metastasis to the liver was noted. Owing to her back pain, an MRI of the spine was done, and it showed pathologic compression fractures at T7, T8, T10, with extensive and diffuse osseous metastatic disease involving the lumbar and thoracic vertebrae (Figures 1D, 1E). Follow-up upper and lower endoscopies did not show any primary GI malignancy. Gastric specimens obtained during endoscopy were significant only for chronic gastritis without any evidence of linitis plastica. A summary of the immunostaining profile of the biopsied specimens from various metastatic sites is shown in Table 2 and representative stained images are shown in Figures 2A-2D.

**Table 2:** Summary of immunostaining results from the biopsied specimen *Weak staining observed; ¶only a few tumor cells stained positive. CK20, Keratin 20; CDX2, caudal type homeobox 2; CK7, keratin 7; PAX8, paired box 8; ER, estrogen receptor; TTF-1, transcription termination factor 1; GATA3, GATA binding protein 3;  WT1, Wilms' tumor protein 1; p40, P protein subunit p40.

Biopsy location	Immunophenotype	Conclusion
Retroperitoneal lymph node	+ MOC31, + CK20, and + CDX2; - CK7, - PAX8, and - ER	Consistent with adenocarcinoma of colorectal origin
Left adnexal mass Right adnexal mass Anterior and posterior cervix	+ CK20 and + CDX2, + Napsin A^*^ and + synaptophysin^*^; - CK7, - TTF-1, - GATA3, - PAX8, - ER, - WT1, - chromogranin, and - p40	Consistent with colorectal or other GI origin. Metastatic adenocarcinoma with signet ring-cell features, rather than primary ovarian mucinous carcinoma
Bone, T7 vertebral Bone, T8 vertebral Bone, T10 vertebral	The tumor tissue is highlighted by pancytokeratin AE1/AE3 + CK20, + CDX2, and + CK7^¶^; - TTF1, - Napsin A, - PAX8, - synaptophysin, - p40, - ER, and - GATA3.	Poorly differentiated colorectal adenocarcinoma, extensively involving the marrow (replacing approximately 50% of marrow spaces)

 

Palliative radiotherapy was initiated and the patient was discharged with the plan for chemotherapy in the outpatient setting.

## Discussion

CEA is the tumor marker associated with colorectal carcinoma (CRC), and it has a poor diagnostic ability for CRC due to overlap with benign disease and low sensitivity for early-stage disease [3,4]. A meta-analysis showed that the pooled sensitivity and specificity of CEA for diagnosing CRC was 46% (95% CI 0.45-0.47) and 89% (95% CI 0.88-0.92), respectively [4]. No other routinely used tumor marker has shown better diagnostic sensitivity and specificity; for example, CA 19-9 showed a pooled sensitivity of 30% (95% CI 0.28-0.32). For these reasons, none of these markers are used as screening or diagnostic tests for CRC. Thus, to date, the use of CEA has been limited to monitoring response to treatment in patients with confirmed CRC [5]. However, the literature provides no guidelines concerning the diagnostic utility of profound elevations of CEA, such as in cases where levels are >20 times the upper limit. As highlighted in our case, such profound CEA elevation is indicative of colorectal cancer until proven otherwise. Besides, in cases where a primary GI tumor cannot be located after upper endoscopy and colonoscopy are performed, profound serum CEA elevation can be of added diagnostic use.

The initial CEA level observed in our patient was markedly elevated at 340.8 (normal range 0.0-3.0 ng/mL), significantly higher than the elevated levels reported in benign conditions such as gastric duplication cysts and metabolic syndrome [6,7]. Thus, when profound CEA levels are noted in serum, as in our case, they most likely represent true tumoral levels, which in the presence of associated findings such as a pelvic mass on CT imaging, indicate malignancy even before a final diagnosis by histopathological analysis is made [8].

## Conclusions

Here, we have presented a case of profound serum CEA elevation and evidence of metastatic adenocarcinoma on CT imaging but with no identified primary GI tumor following upper endoscopy and colonoscopy. Possible reasons for an absent GI primary tumor include complete dislodgement and seeding of the primary tumor into another pelvic organ different from the original tumor site; a primary tumor which, after metastasis, was eliminated by the body’s defenses; or a primary colorectal tumor that remained diminutive, thus escaping detection by endoscopy. These underlying reasons remain speculative at best. However, our case highlights an additional use of profound CEA elevations under such circumstances.
